# Dendritic cells mediate the anti-inflammatory action of omega-3 long-chain polyunsaturated fatty acids in experimental autoimmune uveitis

**DOI:** 10.1371/journal.pone.0219405

**Published:** 2019-07-23

**Authors:** Sho-Hei Uchi, Ryoji Yanai, Masaaki Kobayashi, Makoto Hatano, Yuka Kobayashi, Chiemi Yamashiro, Tomohiko Nagai, Kazuo Tokuda, Kip M. Connor, Koh-Hei Sonoda, Kazuhiro Kimura

**Affiliations:** 1 Department of Ophthalmology, Yamaguchi University Graduate School of Medicine, Ube City, Yamaguchi, Japan; 2 Angiogenesis Laboratory, Department of Ophthalmology, Massachusetts Eye and Ear Infirmary, Harvard Medical School, Cambridge, Massachusetts, United States of America; 3 Department of Ophthalmology, Kyushu University Graduate School of Medical Sciences, Fukuoka City, Fukuoka, Japan; Henan University, CHINA

## Abstract

We previously showed that dietary omega (ω)–3 long-chain polyunsaturated fatty acids (LCPUFAs) suppress inflammation in mice with experimental autoimmune uveitis (EAU). We have now investigated the role of antigen presenting cells (APCs) in this action of ω-3 LCPUFAs. C57BL/6 mice were fed a diet supplemented with ω-3 or ω-6 LCPUFAs for 2 weeks, after which splenocytes were isolated from the mice and cocultured with CD4^+^ T cells isolated from mice with EAU induced by injection of a human interphotoreceptor retinoid-binding protein peptide together with complete Freund’s adjuvant. The proliferation of and production of interferon-γ and interleukin-17 by T cells from EAU mice in vitro were attenuated in the presence of splenocytes from ω-3 LCPUFA–fed mice as compared with those from mice fed ω-6 LCPUFAs. Splenocyte fractionation by magnetic-activated cell sorting revealed that, among APCs, dendritic cells (DCs) were the target of ω-3 LCPUFAs. Adoptive transfer of DCs from mice fed ω-3 LCPUFAs attenuated disease progression in EAU mice as well as the production of pro-inflammatory cytokines by T cells isolated from these latter animals. The proliferation of T cells from control Balb/c mice was also attenuated in the presence of DCs from ω-3 LCPUFA–fed mice as compared with those from ω-6 LCPUFA–fed mice. Furthermore, T cell proliferation in such a mixed lymphocyte reaction was inhibited by prior exposure of DCs from mice fed an ω-6 LCPUFA diet to ω-3 LCPUFAs in vitro. Our results thus suggest that DCs mediate the anti-inflammatory action of dietary ω-3 LCPUFAs in EAU.

## Introduction

Uveitis is a sight-threatening intraocular inflammatory disease. Inflammatory attacks in patients with refractory uveitis can result in irreversible damage to the retina and optic nerve that leads to significant loss of vision. The overproduction of pro-inflammatory cytokines such as tumor necrosis factor (TNF)–α and interleukin (IL)–6 has been found to be associated with inflammatory attacks in uveitis [1]. Types of noninfectious uveitis include Vogt-Koyanagi-Harada disease—a systemic autoimmune disorder that affects pigmented tissues of the body, most prominently the eyes—and sarcoidosis, a disease that can affect any organ and is characterized by the abnormal accumulation of inflammatory cells in granulomas.

The main treatment for noninfectious uveitis is topical or systemic corticosteroid administration. However, long-term corticosteroid treatment is associated with adverse effects such as glaucoma, cataracts, and systemic complications including diabetes, infection, psychological disorders, hypertension, obesity, and osteoporosis [2, 3]. More specific therapies, including the administration of TNF blockers, have recently been introduced for uveitis treatment. However, such agents are not necessarily as effective as corticosteroids, not all patients respond to them, and they are expensive and not devoid of side effects. There is thus a need for new safe and effective treatments for patients with noninfectious uveitis.

The adaptive immune system is essential for defense against infection and tumors, but it can also give rise to autoimmune disease. Antigen presenting cells (APCs)—including dendritic cells (DCs), macrophages, and B lymphocytes—present foreign antigens to naive helper and killer T cells in secondary lymphoid organs and thereby initiate an adaptive immune response. However, stimulation of naive T cells—in particular, T helper (Th) 1 and Th17 cells—by APCs can also elicit inflammation in experimental autoimmune uveitis (EAU) [4]. Conversely, APCs can have immunosuppressive effects mediated by the induction of T cell anergy in the absence of costimulatory molecules or by modulation of the tolerogenic function of regulatory T (Treg) cells in autoimmune disease. They thus have therapeutic potential for the treatment of uveitis as well as of other inflammatory or autoimmune disorders such as multiple sclerosis, neuromyelitis optica, type 1 diabetes, rheumatoid arthritis, and Crohn’s disease [5].

Natural products that may have an efficacy similar to that of conventional drugs but without as many side effects offer a promising approach to the treatment of various conditions. Omega-3 long-chain polyunsaturated fatty acids (ω-3 LCPUFAs), including docosahexaenoic acid (DHA) and eicosapentaenoic acid (EPA), are components of membrane phospholipids and contribute to the maintenance of cell membrane structure and fluidity, intracellular signaling, and cell-cell interactions. They are essential nutrients found primarily in marine fish and fish oil and are also available as dietary supplements. Omega-3 LCPUFAs have been shown to suppress inflammation [6, 7] and neovascularization [8–10] in animal models as well as to protect against the onset of clinically apparent rheumatoid arthritis [11, 12]. Fish, fish oil, or ω-3 LCPUFAs also appear to have beneficial effects on disease activity and pain in individuals with rheumatoid arthritis [13, 14]. Furthermore, studies have suggested that ω-3 LCPUFAs may lower the risk for development of chronic diseases such as heart disease and cancer [15–17].

The anti-inflammatory action of ω-3 LCPUFAs is mediated by the attenuation of effector T cell function both directly and indirectly through effects on APCs such as DCs and macrophages [18, 19]. We have previously shown that dietary intake of ω-3 LCPUFAs suppressed inflammation in association with inhibition of Th1 and Th17 cell function in EAU [7], suggesting that the administration of ω-3 LCPUFAs might be effective for amelioration of inflammation in uveitis patients. We have now investigated the mechanism underlying this anti-inflammatory action of ω-3 LCPUFAs in EAU. In particular, we examined the possible role of APCs in the effects of these fatty acids on T cell differentiation.

## Materials and methods

### Animals and materials

Pathogen-free female C57BL/6 or Balb/c mice at 6 to 8 weeks of age were obtained from Chiyoda Kaihatsu (Tokyo, Japan). Mice were maintained in cages, with at most five animals per cage, at a temperature of 22° to 24°C and relative humidity of 50% to 70% and with a 12-h-light, 12-h-dark cycle (lights on from 8:00 a.m. to 8:00 p.m.). The condition of the animals was monitored at least once a day, and they were killed by cervical dislocation. The mice were treated in accordance with the ARVO (The Association for Research in Vision and Ophthalmology) Statement for the Use of Animals in Ophthalmic and Vision Research, and this study was approved by the Animal Care and Use Committee of Yamaguchi University. Animal chow in which fish product was replaced with an equal number of calories from sunflower oil was obtained from Oriental Yeast (Tokyo, Japan) and was supplemented with 1% DHA and 1% EPA (Lotriga, Takeda, Japan) as ω-3 LCPUFAs or with 1% X and 1% Y (Lotriga) as ω-6 LCPUFAs as described previously [7].

Magnetic-activated cell sorting (MACS) systems for macrophages, pan-DCs, and CD4^+^ T cells were obtained from Miltenyi Biotec (Auburn, CA). Antibodies to CD3, to CD19, to CD11c, and to F4/80 for flow cytometry were obtained from Abcam (Cambridge, MA). Human interphotoreceptor retinoid-binding protein peptide 1–20 (hIRBP(1–20)) was obtained from Scrum (Tokyo, Japan), complete Freund’s adjuvant containing *Mycobacterium tuberculosis* H37Ra at 6 mg/ml was from Difco (Detroit, MI), EPA (*cis*-5,8,11,14,17-eicosapentaenoic acid, ≥99%) and DHA (*cis*-4,7,10,13,16,19-docosahexaenoic acid, ≥98%) in liquid form were from Sigma-Aldrich (St. Louis, MO), and [^3^H]thymidine was from Japan Radioisotope Association (Tokyo, Japan).

### Preparation of APCs for coculture from mice fed an ω-3 LCPUFA diet

C57BL/6 mice were fed chow supplemented with ω-3 LCPUFAs (ω-3 group) or ω-6 LCPUFAs (ω-6 group) for 2 weeks, with the chow provided fresh each day so as to prevent fatty acid oxidation. The mice were then killed, the spleen was removed and dissociated, and red blood cells in the resulting cell suspension were lysed. The remaining splenocytes were incubated with mitomycin C (50 μg/ml) in phosphate-buffered saline for 20 min at 37°C and were then washed three times with RPMI 1640 medium supplemented with 2% penicillin-streptomycin and 10% fetal bovine serum before fractionation. APC subsets were isolated from the spleen cells with the use of MACS kits. The cells were passed first through a column containing MicroBeads conjugated with antibodies to F4/80 in order to isolate macrophages and then through a column containing MicroBeads conjugated with antibodies to CD11c and those to mPDCA-1 in order to isolate DCs. The remaining cells were also collected.

### EAU model and isolation of CD4^+^ T cells

The induction of EAU in C57BL/6 mice was performed as described previously [7]. In brief, mice were injected subcutaneously in the foot pad and the inguinal region each with 50 μg and intraperitoneally with 100 μg of hIRBP(1–20) together with complete Freund’s adjuvant containing *M*. *tuberculosis* H37Ra. The mice were killed 17 days later, and the superficial cervical, axillary, brachial, and inguinal lymph nodes were removed for isolation of CD4^+^ T cells on the basis of negative selection by MACS with antibody-conjugated MicroBeads.

### Coculture of CD4^+^ T cells from EAU mice with APCs from ω-3 LCPUFA–fed mice

Isolated CD4^+^ T cells (3 × 10^5^ in 50 μl of RPMI 1640 per well of a 96-well plate) from EAU mice as well as mitomycin C–treated APCs (3 × 10^5^ in 50 μl of RPMI 1640) from mice of the ω-3 or ω-6 groups were cultured together for 48 h at 37°C in the presence of hIRBP(1–20) at 10 μg/ml. The proliferation of T cells was then measured, and the culture supernatants were collected and stored at –80°C for assay of cytokines.

### Mixed lymphocyte reaction in vitro

A mixed lymphocyte reaction (MLR) was performed by seeding CD4^+^ T cells (3 × 10^5^ in 100 μl of RPMI 1640) isolated by MACS from lymph nodes of Balb/c mice in the wells of a round-profile 96-well plate. Mitomycin C–treated allogeneic DCs, macrophages, or other cells (3 × 10^4^ in 100 μl of RPMI 1640) isolated by MACS from the spleen of C57BL/6 mice fed with ω-3 or ω-6 LCPUFAs for 2 weeks were added to each well, and the cultures were incubated for 48 h. T cell proliferation was then measured, and the culture supernatants were collected and stored at –80°C for assay of cytokines. Alternatively, mitomycin C–treated DCs (3 × 10^4^ per well) from C57BL/6 mice of the ω-6 group were incubated for 48 h with 50 μM DHA or EPA, or with dimethyl sulfoxide (DMSO) vehicle, after which the culture supernatants were discarded and the adherent DCs were incubated for an additional 72 h in an MLR with CD4^+^ T cells (6 × 10^5^ per well) isolated from Balb/c mice. T cell proliferation was again measured.

### Assay of the proliferation of cultured T cells

T cell proliferation was measured on the basis of [^3^H]thymidine incorporation. T cells from cocultures with APCs were harvested and incubated for 12 h with 1 μCi of [^3^H]thymidine (5 Ci/mmol) in the wells of a 96-well plate. The cells were then transferred to glass-fiber filters for measurement of [^3^H]thymidine incorporation by scintillation spectroscopy.

### Assay of pro-inflammatory cytokines released by cultured T cells

The concentrations of the pro-inflammatory cytokines interferon (IFN)–γ and IL-17 in culture supernatants were measured with enzyme-linked immunosorbent assay (ELISA) kits (R&D Systems, Minneapolis, MN).

### EAU model with adoptive transfer of DCs

Splenocytes of C57BL/6 mice fed a diet supplemented with ω-3 or ω-6 LCPUFAs for 14 days were subjected to MACS for isolation of DCs. The DCs (2.5 × 10^6^ cells) were injected intraperitoneally into EAU mice at 7 days after hIRBP(1–20) injection.

### Clinical scoring of EAU

Clinical signs of EAU were evaluated at 7, 14, 17, 23, and 28 days after disease induction. Mice were anesthetized by intraperitoneal injection of a mixture of ketamine (90 mg/kg) and xylazine (10 mg/kg), and their pupils were dilated by topical instillation of 0.5% tropicamide and 0.5% phenylephrine hydrochloride ophthalmic solutions for examination by indirect funduscopy. The clinical score for EAU was determined from the photographs by retinal specialists in a blinded manner and according to a scale of 0 to 5: 0, no evidence of inflammation; 1, focal vasculitis or spotted soft exudate of less than five spots; 2, linear vasculitis or spotted soft exudate within half of the retina; 3, linear vasculitis or spotted soft exudate encompassing more than half of the retina; 4, retinal hemorrhage as well as severe vasculitis or spotted soft exudate; and 5, exudative retinal detachment or subretinal hemorrhage.

### Histological scoring of EAU

Histological evaluation of EAU was performed as described previously [7]. Mice were killed 17 days after induction of EAU (10 days after transfer of DCs), and the eyeballs were removed and fixed in TB-fix [20] for preparation of paraffin-embedded sections (thickness of 5 μm) in the papillary–optic nerve plane. The sections were stained with hematoxylin-eosin, and EAU was scored in each eye on a scale of 0 to 3: 0, no disease and normal retinal architecture; 1, mild infiltration in the uvea, vitreous, and retina as well as the presence of retinal folds, vasculitis, and one small granuloma; 2, moderate infiltration in the uvea, vitreous, and retina as well as the presence of retinal folds, vasculitis, focal shallow detachments, small granulomas, and focal photoreceptor cell damage; 3, moderate to severe infiltration in the uvea, vitreous, and retina as well as the presence of extensive retinal folding with large detachments, subretinal neovascularization, moderate photoreceptor cell damage, and medium-size granulomatous lesions.

### Assay of cytokine release from T cells of EAU mice after adoptive transfer of DCs

At 17 days after disease induction (10 days after DC transfer), CD4^+^ T cells (3 × 10^5^ per well of a 96-well plate) isolated from EAU model mice by MACS were cultured for 24 h in 200 μl of RPMI 1640 medium. The concentrations of cytokines in the culture supernatants were then measured with the use of a Bio-Plex Pro Mouse Cytokine 23-Plex Panel and Bio-Plex Manager software version 4.1.1 (Bio-Rad, Hercules, CA).

### Assay of immune-checkpoint molecule expression in T cells of EAU mice after adoptive transfer of DCs

At 17 days after disease induction (10 days after DC transfer), CD4^+^ T cells were isolated from EAU model mice by MACS and were lysed (2 × 10^7^ cells/ml) in an ice-cold extraction buffer for measurement of programmed cell death (PD)–1 and cytotoxic T lymphocyte–associated protein (CTLA)–4 with ELISA kits (Abcam).

### Statistical analysis

Quantitative data are presented as means ± SD or means ± SEM and were analyzed with the use of Prism version 6 for Windows (GraphPad Software, San Diego, CA). EAU scores were compared between groups with the nonparametric Mann-Whitney U test. Cytokine concentration and cell proliferation data were compared with the Mann-Whitney U test, Sidak’s multiple comparison test, or the Tukey-Kramer multiple comparison test. A *P* value of <0.05 was considered statistically significant.

## Results

### APCs from mice fed ω-3 LCPUFAs suppress Th1 and Th17 cell inflammation in vitro

**Fig 1:**
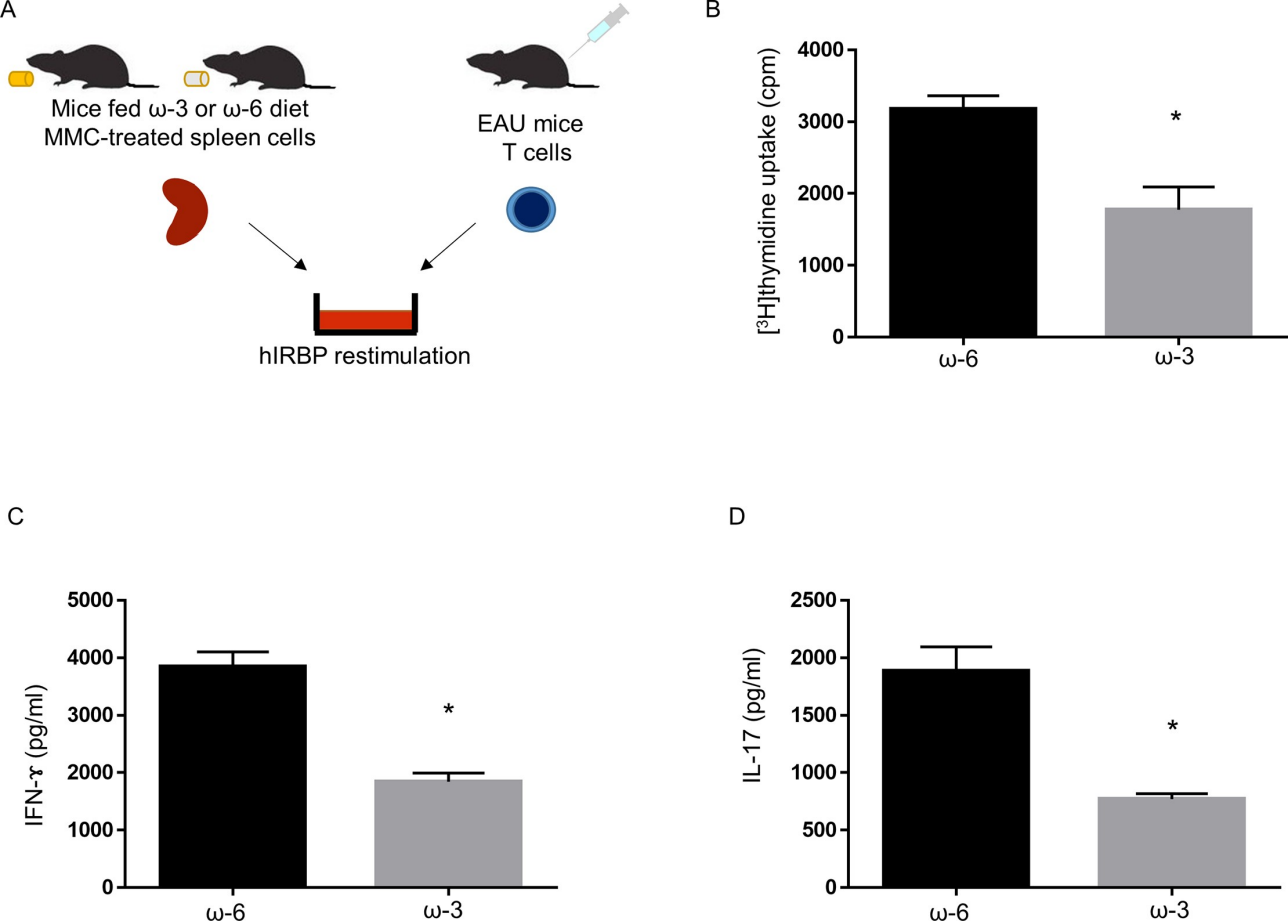
T cell proliferation as well as IFN-γ and IL-17 production in cocultures of CD4^+^ T cells from EAU mice and APCs from mice fed ω-3 or ω-6 LCPUFAs. (A) Experimental scheme. Mitomycin C (MMC)–treated spleen cells (3 × 10^5^) isolated and separately pooled from mice fed a diet supplemented with ω-3 (*n* = 5) or ω-6 (*n* = 5) LCPUFAs for 14 days were cultured for 48 h in the presence of hIRBP(1–20) (10 μg/ml) and with CD4^+^ T cells (3 × 10^5^) isolated and pooled from lymph nodes of EAU mice (*n* = 5) at 17 days after injection of hIRBP(1–20). (B) The proliferation of the T cells was then assessed by measurement of [^3^H]thymidine incorporation. Data are means + SEM from three independent experiments. (C, D) The concentrations of IFN-γ (C) and IL-17 (D) in the coculture supernatants were measured with ELISAs. Data are means + SEM from three independent experiments. *P < 0.05 versus ω-6 LCPUFA diet (Mann-Whitney U test).

To investigate the mechanism underlying the anti-inflammatory action of dietary ω-3 LCPUFAs in EAU, we isolated APCs from the spleen of mice fed for 14 days with a diet supplemented either with these fatty acids or with ω-6 LCPUFAs as a control (Fig 1A). The cells were treated with mitomycin C to inhibit DNA synthesis and cell division and were then cultured together with CD4^+^ T cells isolated from EAU mice at 17 days after disease induction as well as in the presence of hIRBP(1–20) to restimulate the T cells in vitro. The proliferation of the T cells was significantly attenuated in the presence of APCs from mice fed the ω-3 LCPUFA diet compared with that in the presence of those from mice fed the ω-6 LCPUFA diet (Fig 1B). The concentrations of the Th1 cytokine IFN-γ and the Th17 cytokine IL-17 in the coculture supernatants were also markedly lower in the presence of APCs from mice fed the ω-3 LCPUFA diet than in that of those from mice fed the ω-6 LCPUFA diet (Fig 1C and 1D).

### Role of DCs in the suppression of Th1 and Th17 cell inflammation in vitro by spleen cells from mice fed ω-3 LCPUFAs

**Fig 2:**
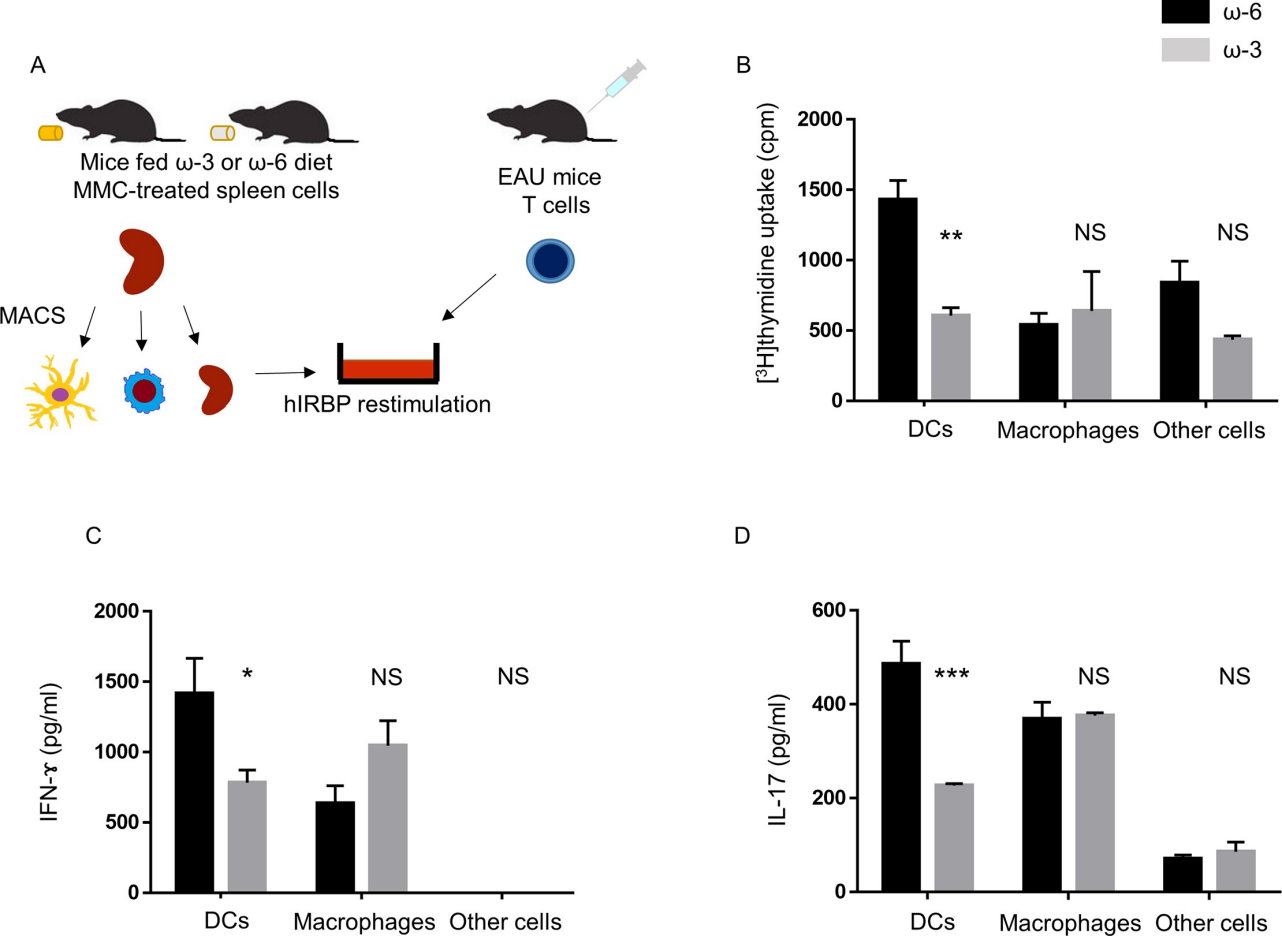
T cell proliferation as well as IFN-γ and IL-17 production in cocultures of CD4^+^ T cells from EAU mice and APC fractions from mice fed ω-3 or ω-6 LCPUFAs. (A) Experimental scheme. Mitomycin C–treated spleen cells isolated and separately pooled from mice fed a diet supplemented with ω-3 (*n* = 5) or ω-6 (*n* = 5) LCPUFAs were fractionated by MACS into DCs, macrophages, and other spleen cells. Each cell fraction (3 × 10^5^ cells) was incubated for 48 h in the presence of hIRBP(1–20) (10 μg/ml) and with CD4^+^ T cells (3 × 10^5^) isolated and pooled from EAU mice (*n* = 5). (B) The proliferation of the T cells was then assessed by measurement of [^3^H]thymidine incorporation. Data are means + SEM from three independent experiments. (C, D) The concentrations of IFN-γ (C) and IL-17 (D) in the coculture supernatants were measured with ELISAs. Data are means + SEM of triplicates from one experiment and are representative of three independent experiments.*P < 0.05, **P < 0.01, ***P < 0.001; NS, not significant versus corresponding value for the ω-6 LCPUFA diet (Sidak’s multiple comparison test).

We next investigated which type of APC from ω-3 LCPUFA–fed mice is responsible for suppression of the proliferation of and cytokine production by T cells from EAU mice. Mitomycin C–treated spleen cells from mice fed diets supplemented with ω-3 or ω-6 LCPUFAs were fractionated by MACS into DCs, macrophages, and other cells (Fig 2A). DCs from ω-3 LCPUFA–fed mice significantly inhibited T cell proliferation (Fig 2B) as well as the production of IFN-γ (Fig 2C) and IL-17 (Fig 2D) in cocultures compared with DCs from mice fed the ω-6 LCPUFA diet. In contrast, these assays revealed no significant differences in the effects of macrophages or other spleen cells between mice of the ω-3 or ω-6 groups. To determine whether dietary intake of ω-3 LCPUFAs might affect the proportions of DCs (CD11c^+^), macrophages (F4/80^+^), T cells (CD3^+^), and B cells (CD19^+^) in the spleen, we examined the expression of cell surface makers by flow cytometry. No differences in the percentages of these cell types were apparent between the spleen of mice fed the ω-3 LCPUFA diet and that of those fed the ω-6 LCPUFA diet (S1 Fig). These results thus suggested that dietary intake of ω-3 LCPUFAs targeted DCs to suppress inflammation mediated by Th1 and Th17 cells in vitro.

### Suppression of inflammation in EAU mice by adoptive transfer of DCs from mice fed ω-3 LCPUFAs

**Fig 3:**
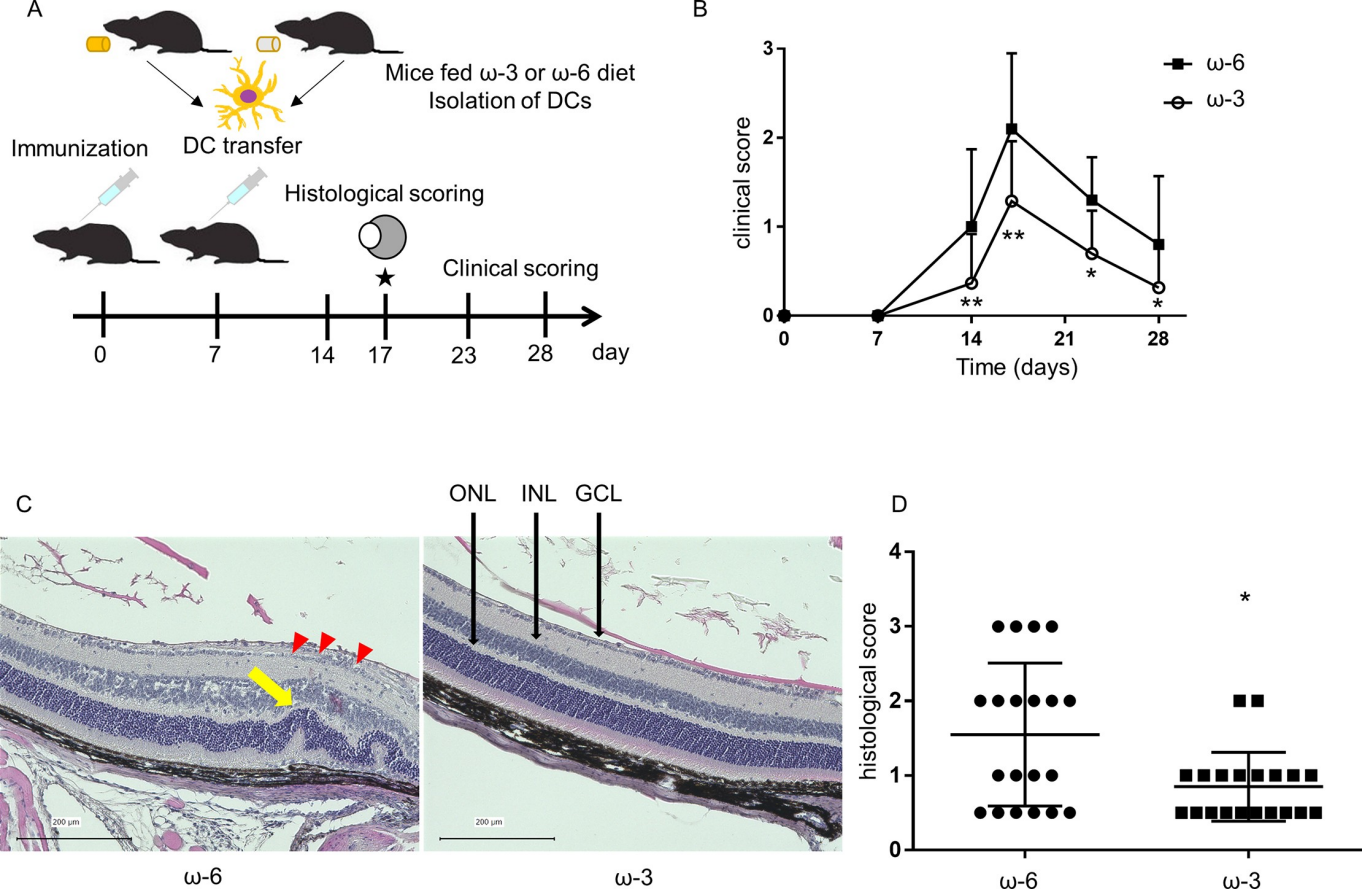
Clinical and histological scoring of EAU in model mice after adoptive transfer of DCs from ω-3 LCPUFA–fed mice. (A) Experimental scheme. At 7 days after disease induction, EAU mice underwent adoptive transfer of DCs (2.5 × 10^6^) isolated and separately pooled from the spleen of mice fed a diet supplemented with ω-3 (*n* = 5) or ω-6 (*n* = 5) LCPUFAs. (B) The clinical score of EAU was determined 7, 14, 17, 23, and 28 days after disease induction. Data are means + SD for both eyes of five EAU mice per group. (C) Hematoxylin-eosin staining of retinal sections of EAU mice at 17 days after disease induction. Red arrowheads indicate inflammatory cells in the retina; yellow arrow indicates a retinal fold. GCL, ganglion cell layer; INL, inner nuclear layer; ONL, outer nuclear layer. Scale bars, 200 μm. (D) Histological score for EAU determined from sections similar to those in (C). Data are shown for individual eyes of 10 EAU mice per group, and the mean ± SD values are also indicated. *P < 0.05, **P < 0.01 versus the corresponding value for the ω-6 LCPUFA diet (Mann-Whitney U test).

We performed both clinical and histological scoring of EAU in model mice that had undergone adoptive transfer of DCs from ω-3 or ω-6 LCPUFA–fed mice at 7 days after disease induction (Fig 3A). The clinical score of EAU at 14 to 28 days after disease induction was significantly lower in the mice that received DCs from ω-3 LCPUFA–fed mice than in those that received DCs from mice of the ω-6 group (Fig 3B). Histological analysis at 17 days after disease induction (when the clinical score was maximal) revealed retinal folds and immune cell infiltration in the eyes of EAU mice that received DCs from ω-6 LCPUFA–fed mice, and that such changes were markedly suppressed in EAU mice that received DCs from ω-3 LCPUFA–fed mice (Fig 3C). The histological score of EAU was thus significantly lower in the mice that received DCs from ω-3 LCPUFA–fed mice (Fig 3D).

### Expression of cytokines and immune-checkpoint molecules in T cells of EAU mice after adoptive transfer of DCs from ω-3 LCPUFA–fed mice

**Fig 4:**
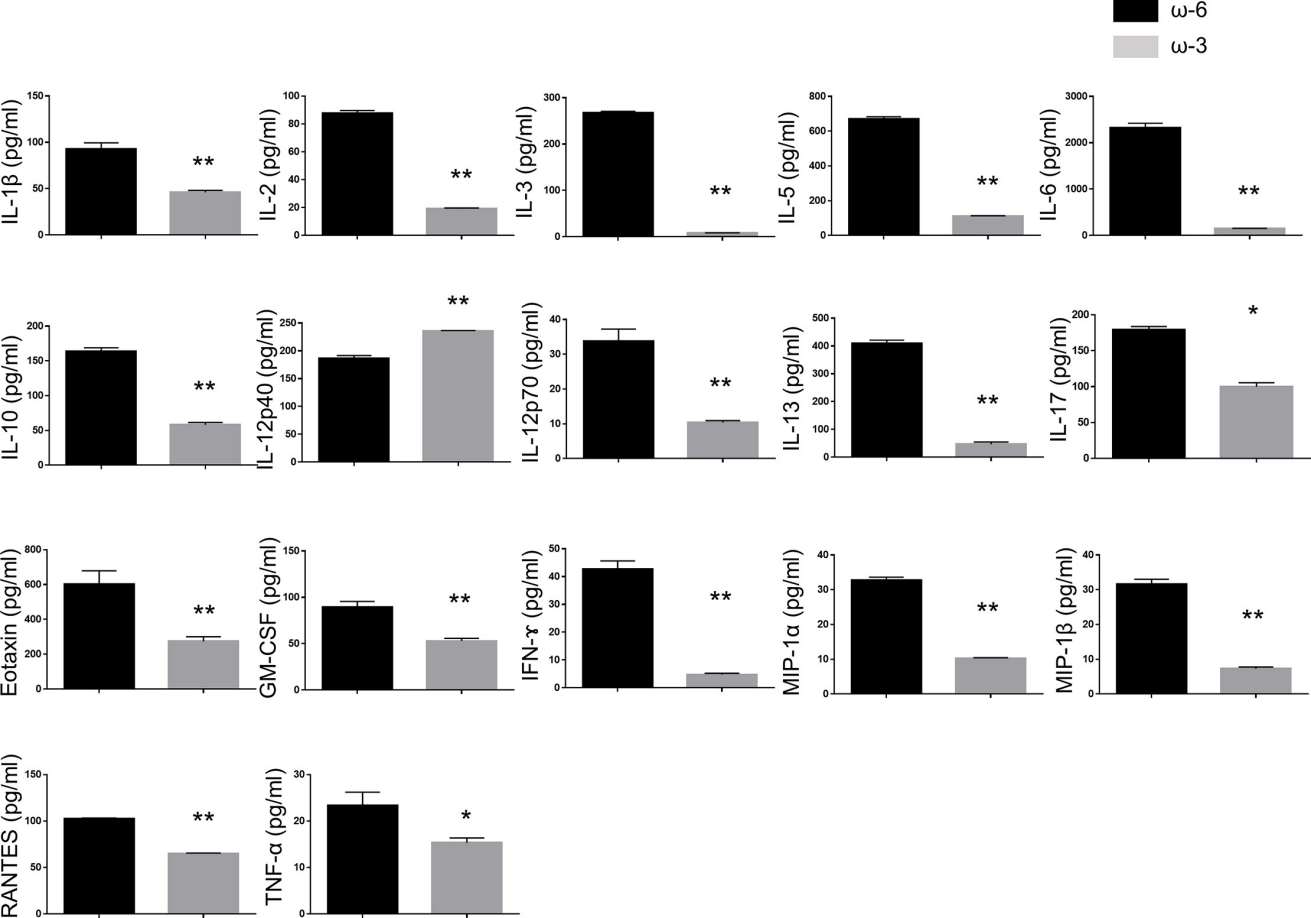
Cytokine production by T cells isolated from EAU mice after adoptive transfer of DCs from ω-3 LCPUFA–fed mice. CD4^+^ T cells isolated from EAU mice at 10 days after the adoptive transfer of DCs from mice fed a diet containing ω-3 (*n* = 5) or ω-6 (*n* = 5) LCPUFAs (17 days after disease induction) were cultured for 24 h, after which the concentrations of the indicated cytokines in the culture supernatants were determined with a multiplex assay. Data are means + SEM for four EAU mice per group. *P < 0.05, **P < 0.01 versus the ω-6 LCPUFA diet (Mann-Whitney U test).

We next examined the production of cytokines by CD4^+^ T cells isolated from EAU mice at 10 days after the adoptive transfer of DCs from mice fed a diet supplemented with ω-3 or ω-6 LCPUFAs. A multiplex assay revealed that the production of pro-inflammatory cytokines including IL-1β, IL-2, IL-3, IL-5, IL-6, IL-10, IL-12 p70, IL-13, eotaxin, granulocyte-macrophage colony-stimulating factor (GM-CSF), IFN-γ, macrophage inflammatory protein (MIP)–1α, MIP-1β, RANTES (regulated on activation, normal T expressed and secreted), and TNF-α was significantly attenuated in T cells from mice that received DCs from ω-3 LCPUFA–fed mice compared with that in those from mice that received DCs from mice in the ω-6 group (Fig 4). In contrast, the production of IL-12 p40 by T cells was increased by adoptive transfer of DCs from ω-3 LCPUFA–fed mice. ELISAs also revealed that expression of the immune-checkpoint molecules PD-1 and CTLA-4 in T cells did not differ between EAU mice that received DCs from mice with or without dietary exposure to ω-3 LCPUFAs (S2 Fig).

### Effects of DCs from ω-3 LCPUFA–fed mice on T cell proliferation and cytokine production in an MLR

**Fig 5:**
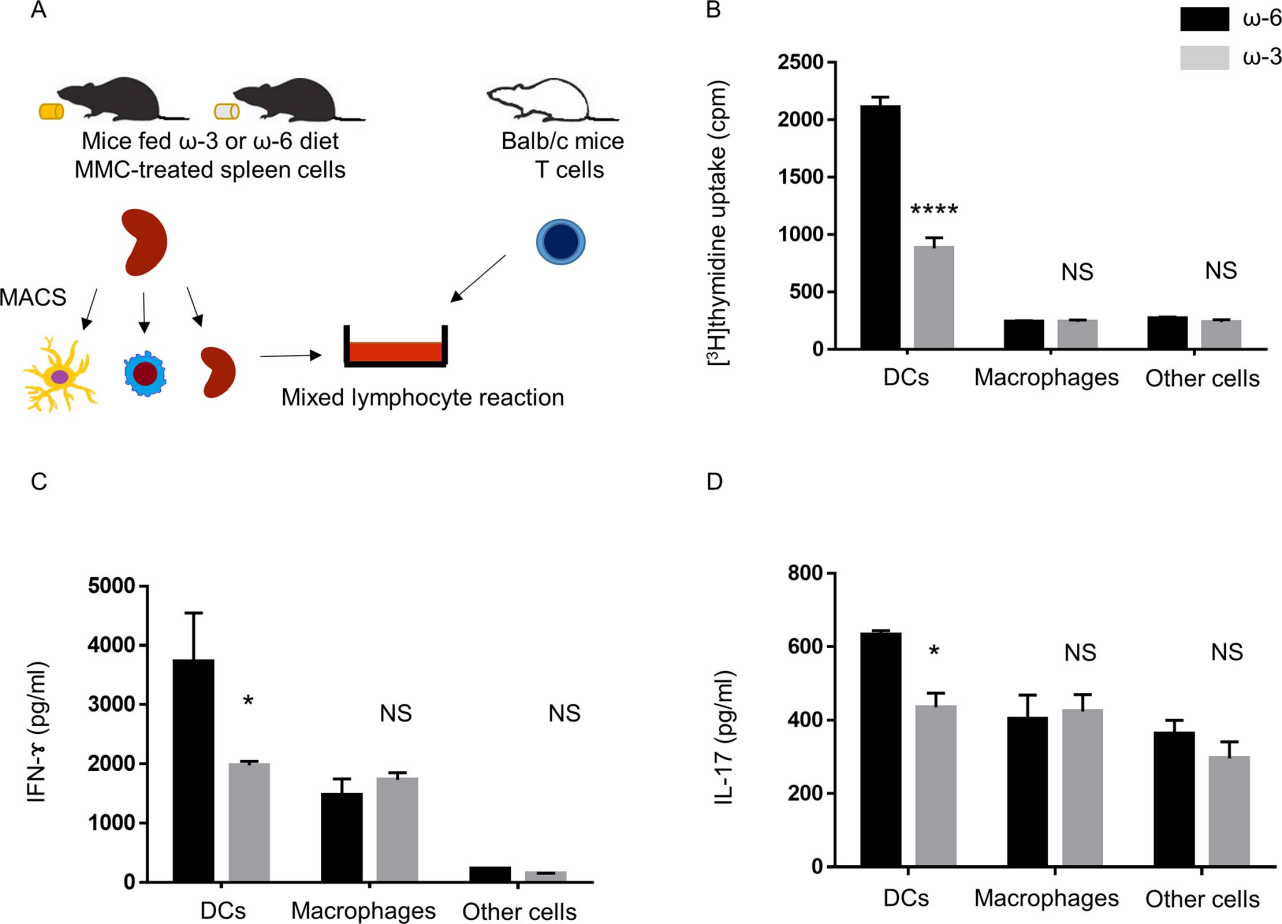
Effects of DCs from mice fed an ω-3 LCPUFA diet on T cell proliferation and cytokine production in an MLR. (A) Experimental scheme. Mitomycin C–treated spleen cells isolated and separately pooled from C57BL/6 mice fed a diet supplemented with ω-3 (*n* = 5) or ω-6 (*n* = 5) LCPUFAs were fractionated into DCs, macrophages, and other cells. Each of these cell fractions (3 × 10^4^ cells) was then incubated for 48 h with CD4^+^ T cells (3 × 10^5^) isolated and pooled from Balb/c mice (*n* = 5). (B) The proliferation of the T cells was then assessed by measurement of [^3^H]thymidine incorporation. (C, D) The concentrations of IFN-γ (C) and IL-17 (D) in the coculture supernatants were measured with ELISAs. Data are means + SEM from three independent experiments. *P < 0.05, **** *P* < 0.0001, NS versus the corresponding value for the ω-6 LCPUFA diet (Sidak’s multiple comparison test).

We examined the effects of mitomycin C–treated cells isolated from the spleen of C57BL/6 mice fed a diet containing or lacking ω-3 LCPUFAs on the proliferation and cytokine production by CD4^+^ T cells from Balb/c mice in an MLR (Fig 5A). T cell proliferation (Fig 5B) as well as IFN-γ (Fig 5C) and IL-17 (Fig 5D) production were significantly attenuated in the MLR with DCs from mice of the ω-3 group compared with those apparent in the presence of DCs from mice of the ω-6 group. In contrast, the extent of T cell proliferation or cytokine production did not differ significantly in the MLRs performed with macrophages or other spleen cells from mice fed with or without ω-3 LCPUFAs.

### Direct inhibition by ω-3 LCPUFAs of the ability of DCs to activate T cells in an MLR

**Fig 6:**
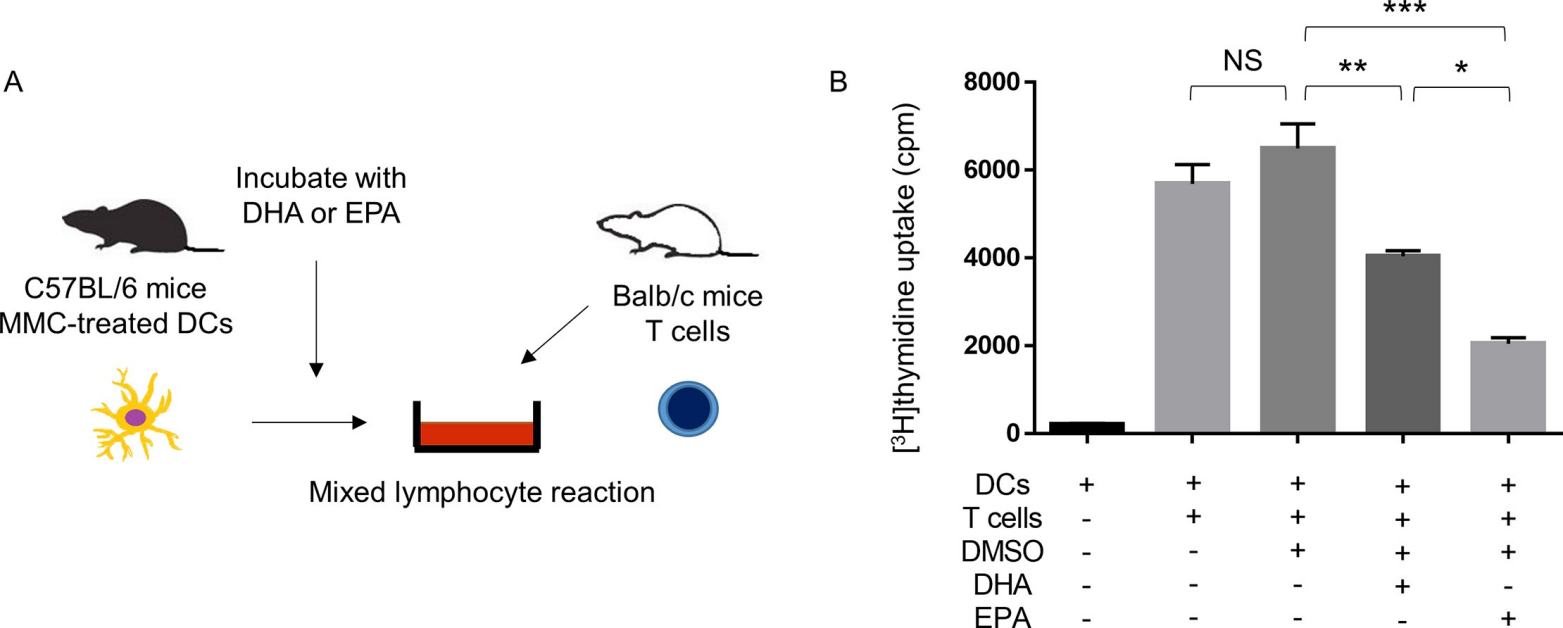
Direct inhibition by ω-3 LCPUFAs of the ability of DCs to stimulate T cell proliferation in an MLR. (A) Experimental scheme. Mitomycin C–treated DCs (3 × 10^4^) isolated and pooled from C57BL/6 mice fed an ω-3 LCPUFA–free diet (*n* = 5) were cultured in the presence of DHA or EPA (50 μM) or of DMSO vehicle for 48 h, after which the cells were cocultured for 72 h with CD4^+^ T cells (6 × 10^5^) isolated and pooled from Balb/c mice (*n* = 5) in an MLR. (B) The proliferation of the T cells was then assessed by measurement of [^3^H]thymidine incorporation. Data are means + SEM from two independent experiments. *P < 0.05, ** *P* < 0.01, ***P < 0.001, NS (Tukey-Kramer multiple comparison test).

Finally, we examined the effect of direct exposure of DCs to ω-3 LCPUFAs in vitro on the stimulatory effect of DCs on T cell proliferation in an MLR (Fig 6A). Exposure of mitomycin C–treated DCs from C57BL/6 mice fed an ω-3 LCPUFA–free diet to DHA or EPA (50 μM) for 48 h before their incubation with CD4^+^ T cells from Balb/c mice in an MLR resulted in significant attenuation of the stimulatory effect of the DCs on T cell proliferation (Fig 6B). The inhibitory effect of EPA was significantly greater than that of DHA.

## Discussion

Our results have shown that the proliferation of as well as Th1 and Th17 cytokine production by CD4^+^ T cells from EAU mice in vitro were attenuated in the presence of DCs from mice fed a diet supplemented with ω-3 LCPUFAs. Furthermore, adoptive transfer of DCs from ω-3 LCPUFA–fed mice attenuated disease progression in EAU mice as well as suppressed the production of pro-inflammatory cytokines by T cells isolated from these latter mice. These anti-inflammatory effects of ω-3 LCPUFAs were also apparent in an MLR performed with DCs isolated from ω-3 LCPUFA–fed mice or with DCs directly exposed to DHA or EPA in vitro. These observations have thus provided insight into the mechanism by which ω-3 LCPUFAs attenuate inflammation in uveitis and may serve as the basis for the development of new clinical treatment.

The potential of dietary intervention as a preventive or ameliorative treatment for inflammatory disease has gained attention in recent years, with such an approach having the benefit of reducing both drug costs and side effects. Several studies have demonstrated the efficacy of ω-3 LCPUFAs—including α-linolenic acid, DHA, and EPA—as anti-inflammatory agents in various diseases [21, 22]. We have previously shown that dietary intake of ω-3 LCPUFAs suppressed uveitis in mice in association with inhibition of Th1 and Th17 cell function [7]. Our present results now implicate DCs as a mediator of the anti-inflammatory action of dietary ω-3 LCPUFAs in EAU.

Activation of immune cells, including DCs, plays an essential role in inflammatory or autoimmune diseases. Given that dietary ω-3 LCPUFAs have been shown to have a protective action in models of such diseases including polymicrobial sepsis, periodontitis, peritonitis, and colitis [23–27], an understanding of their effects on immune cells will contribute to the future development of these fatty acids or their derivatives as anti-inflammatory therapeutic agents. DHA has been shown to prevent the maturation of DCs and thereby to inhibit the differentiation of Th1 and Th17 effector cells [6], consistent with our present finding that mouse spleen-derived DCs mediate the anti-inflammatory action of ω-3 LCPUFAs in EAU. We found that ω-3 LCPUFAs did not affect the cellular composition of the spleen in terms of the proportions of T cells, B cells, DCs, and macrophages.

In vitro experiments with human monocyte-derived DCs showed that ω-3 LCPUFAs prevent lipopolysaccharide-induced up-regulation both of the expression of major histocompatibility complex type II and costimulatory (CD80, CD86, CD40) molecules and of cytokine secretion [28]. Cytokines released by activated DCs are essential for T cell differentiation, with IL-12 p70 promoting Th1 cell differentiation and with IL-6 and transforming growth factor–β promoting Th17 cell differentiation [29, 30]. We have now found that dietary ω-3 LCPUFAs acting via adoptively transferred DCs markedly inhibited IL-12 p70 and IL-6 production by T cells from EAU mice. These results thus suggest that ω-3 LCPUFAs suppress Th1 and Th17 cytokine production by CD4^+^ T cells through DCs. We also found that ω-3 LCPUFAs, again acting via DCs, suppressed the production not only of pro-inflammatory cytokines but also of the anti-inflammatory cytokine IL-10. Dietary ω-3 LCPUFAs were previously shown to suppress the production of IL-10 by macrophages measured ex vivo [31]. The anti-inflammatory effects of ω-3 LCPUFAs appear to outweigh their pro-inflammatory effects, however, at least in EAU.

Cell culture and animal studies have found that ω-3 LCPUFAs inhibit the proliferation of and IL-2 production by T cells [32–36]. In the present study, DCs exposed to ω-3 LCPUFAs either in vivo or in vitro suppressed T cell proliferation, suggesting that exposure of DCs to ω-3 LCPUFAs attenuates inflammation mediated by T cells. However, the intracellular signaling pathways activated by ω-3 LCPUFAs and their target genes remain largely unknown. Mediators shown to be derived from ω-3 LCPUFAs include prostaglandins, leukotrienes, resolvins, peroxisome proliferator–activated receptor (PPAR)–γ ligands, and cytochrome P450 metabolites [8, 37, 38]. Further studies are needed to identify which lipid mediators are responsible for the DC-dependent anti-inflammatory action of ω-3 LCPUFAs in EAU.

## Supporting information

S1 Fig Lack of effect of dietary intake of ω-3 LCPUFAs on the cellular composition of the spleen. Spleen cells from mice fed a diet supplemented with ω-3 or ω-6 LCPUFAs for 14 days were stained (or not) with antibodies to CD3 (T cells), to CD19 (B cells), to CD11c (DCs), or to F4/80 (macrophages) and were then analyzed by flow cytometry.
(TIF)

S2 Fig Expression of immune-checkpoint molecules in T cells isolated from EAU mice after adoptive transfer of DCs from ω-3 LCPUFA–fed mice. CD4^+^ T cells isolated from EAU mice at 10 days after the adoptive transfer of DCs from C57BL/6 mice fed a diet containing ω-3 or ω-6 LCPUFAs (17 days after disease induction) were lysed and assayed for the expression of PD-1 and CTLA-4 with ELISAs. Data are means + SEM for five EAU mice per group. NS versus the ω-6 LCPUFA diet (Mann-Whitney U test).
(TIF)

S1 Dataset Minimal data set. A summary of minimal data set. Mean, S.D., Statistics method used and P value for each Figs 1–6 were summarized.
(XLSX)
