# Correction: Dendritic cells mediate the anti-inflammatory action of omega-3 long-chain polyunsaturated fatty acids in experimental autoimmune uveitis

**DOI:** 10.1371/journal.pone.0231277

**Published:** 2020-03-27

**Authors:** Sho-Hei Uchi, Ryoji Yanai, Masaaki Kobayashi, Makoto Hatano, Yuka Kobayashi, Chiemi Yamashiro, Tomohiko Nagai, Kazuhiro Tokuda, Kip M. Connor, Koh-Hei Sonoda, Kazuhiro Kimura

The eighth author's name is spelled incorrectly. The correct name is: Kazuhiro Tokuda.
