# Supplementary figures and images for: Dendritic cells mediate the anti-inflammatory action of omega-3 long-chain polyunsaturated fatty acids in experimental autoimmune uveitis
Source: PLoS One. 2019 Jul 23;14(7):e0219405. doi: 10.1371/journal.pone.0219405 (PMC6650034; doi:10.1371/journal.pone.0219405)

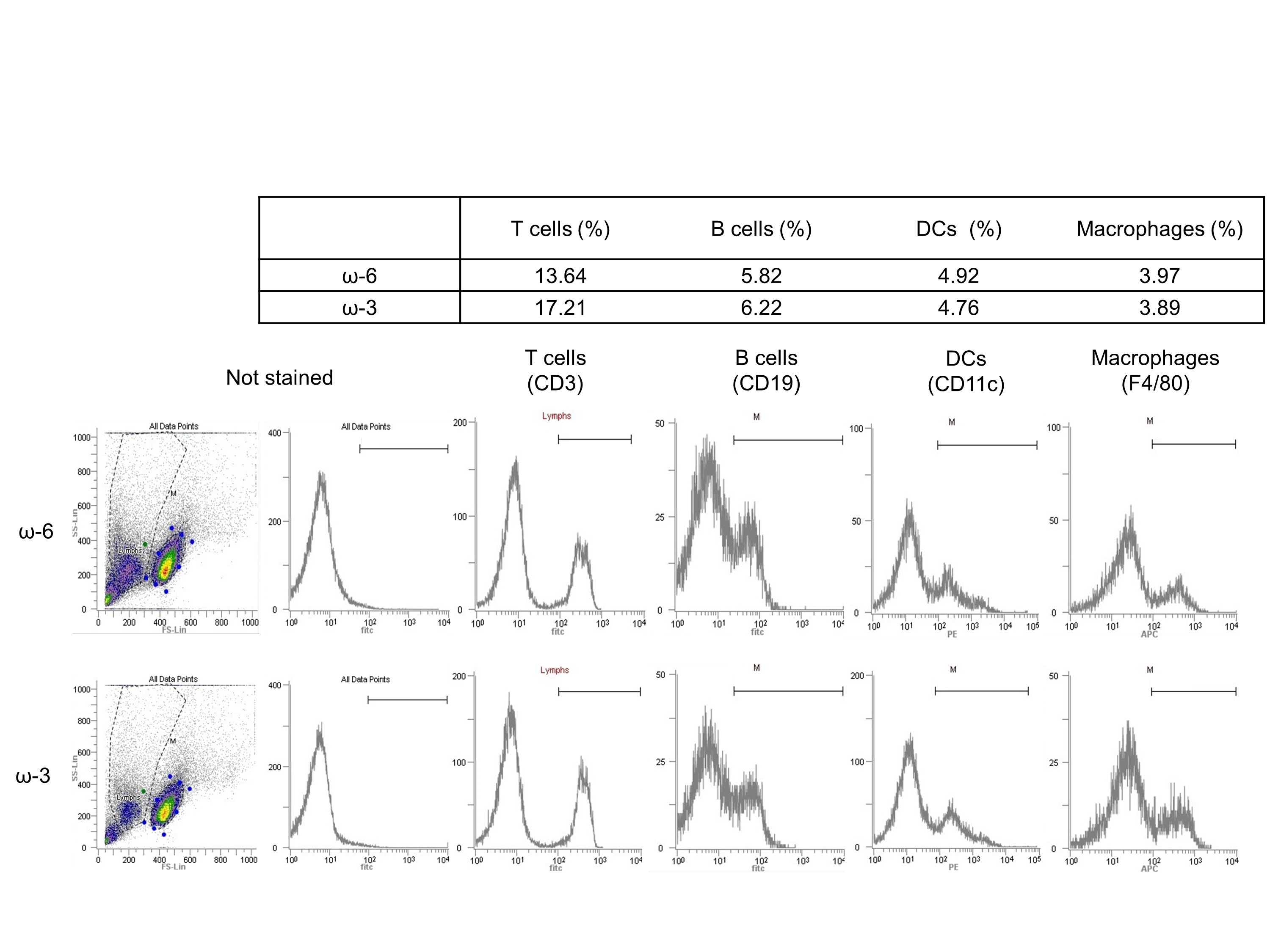

Supplement: S1 Fig — Spleen cells from mice fed a diet supplemented with ω-3 or ω-6 LCPUFAs for 14 days were stained (or not) with antibodies to CD3 (T cells), to CD19 (B cells), to CD11c (DCs), or to F4/80 (macrophages) and were then analyzed by flow cytometry. (TIF) [file pone.0219405.s001.TIF]

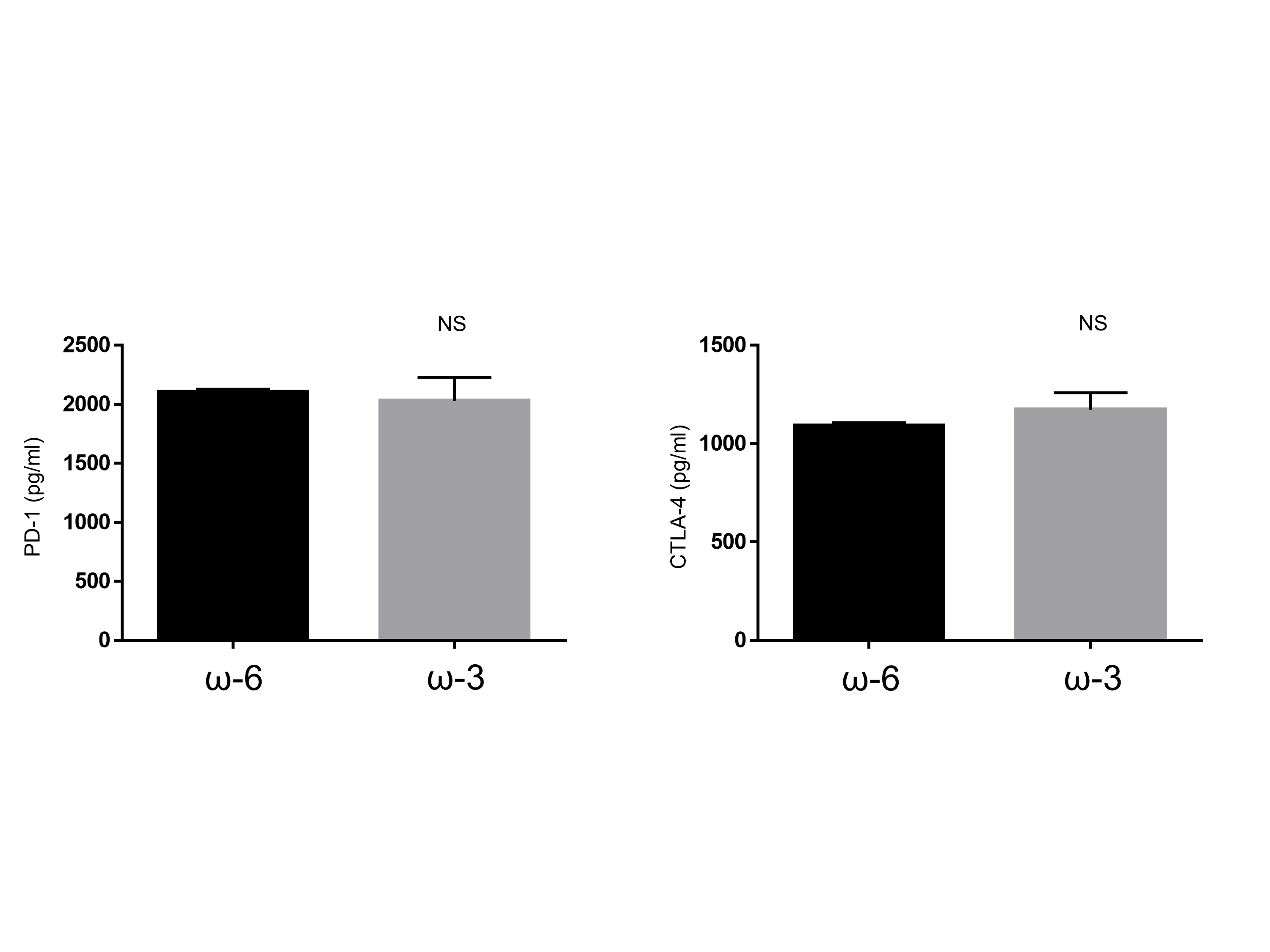

Supplement: S2 Fig — CD4+ T cells isolated from EAU mice at 10 days after the adoptive transfer of DCs from C57BL/6 mice fed a diet containing ω-3 or ω-6 LCPUFAs (17 days after disease induction) were lysed and assayed for the expression of PD-1 and CTLA-4 with ELISAs. Data are means + SEM for five EAU mice per group. NS versus the ω-6 LCPUFA diet (Mann-Whitney U test). (TIF) [file pone.0219405.s002.TIF]
